# Thiamine deficiency affects glucose transport and β‐oxidation in rats

**DOI:** 10.1111/jpn.13146

**Published:** 2019-07-01

**Authors:** Mikołaj Antoni Gralak, Bogdan Dębski, Małgorzata Drywień

**Affiliations:** ^1^ Department of Physiological Sciences, Faculty of Veterinary Medicine Warsaw University of Life Sciences‐SGGW Warsaw Poland; ^2^ Department of Human Nutrition, Faculty of Human Nutrition and Consumer Sciences Warsaw University of Life Sciences‐SGGW Warsaw Poland

**Keywords:** thiamine deficiency glucose uptake β‐oxidation blood cells

## Abstract

Thiamine is recognized as a cofactor for many enzymes involved in intermediary metabolism responsible for energy production. Animal model of thiamine deficiency (TD) included direct evaluation of glucose uptake by estimation of ^3^H‐deoxyglucose transport across red blood cells membranes and β‐oxidation of fatty acids in isolated leucocytes. Feeding of animals with the thiamine‐deficient diet (0.018 mg/kg diet) for 30 days resulted in disturbances in energy production. The thiamine intake was limited not only by vitamin B_1_ deficiency in the diet, but also by time‐dependent drop of feed consumption by rats fed this diet. At the end of experiment, diet consumption in this group of rats was 52% lower than in the control group. This was accompanied by low glucose uptake by erythrocytes of rats suffering vitamin B_1_ deficiency for longer time. At the end of experimental period, glucose uptake was over 2 times lower in TD erythrocytes than in control RBC. Such drop of energy production was not compensated by delivery of energy from fatty acid degradation. In leucocytes from TD rats, the β‐oxidation was also suppressed. Observed significant decrease of serum insulin from 2.25 ± 0.25 ng/ml (day 0) to 1.94 ± 0.17 ng/ml (day 30) might have significant impact on observed energy production disorders. The results from this study indicate that the thiamine deficiency significantly reduces feed intake and causes modest abnormalities in glucose and fatty acid utilization.

## 1 INTRODUCTION

Thiamine plays a fundamental role in intermediary metabolism. In the organism, it is converted to thiamine pyrophosphate, which is a cofactor for several enzymes necessary for decarboxylation of α‐ketoacids (pyruvic and α‐ketoglutaric acids), including those formed in transamination of branched‐chain amino acids. It participates in transketolation in pentose cycle. Vitamin B_1_ (thiamine) is an activator of acetylcholine action by inhibition of acetylcholine esterase and acts synergistically with thyroxin and insulin. Moreover, it is a factor regulating insulin secretion by pancreatic cells. Thiamine‐deficient rats exhibit lower insulin level, probably due to decreased glucose oxidation (Ratahanaswami, Pourany, & Sundaresan, 1991). It is also an activator of secretion of gonadotropic hormones in the anterior pituitary.

Thiamine deficiency (TD) causes besides the weight loss, also disorders of nervous system and cardiomyopathy (Hoffman., 2016; Krasicka, Gralak, Sieranska, & Kulasek, 1999). It may decrease the membrane conductance, cause disorders in ATP production (Oliveira, Galan, Ribeiro, & Santos Cruz, 2007), brain failures (Aikawa et al., 1984; Ke, DeGiorgio, Volpe, & Gibson, 2003; Yu et al., 2018) and liver disorders (Hernandez‐Vazquez et al., 2016). Moreover, the TD may result in an increase of the frequency of death of cardiac cells (Zangen & Shainberg, 1997). These symptoms of the TD were reviewed by Gibson et al. (2013), Manzetti, Zhang, and Spoel (2014) and Osiezagha et al. (2013). The final effect of the prolonged severe thiamine deficiency is beriberi disease. The chronic state of the TD contributes to the development of neurodegenerative diseases such as Parkinson's disease, Alzheimer's disease, Huntington's disease and Wernicke–Korsakoff syndrome. Thiamine deficiency may result from the inadequate intake of this vitamin, an increased requirement, an excessive excretion, a high consumption of anti‐thiamine factors or a combination of above‐mentioned factors. Anti‐thiamine compounds include thiaminase I encountered in many raw fish and shellfish and thermostable tannins identified in some teas and betel nuts (Lepak, Kraft, & Vanni, M.J., 2013). Thiamine supplementation is especially important for physically active individuals, who require more energetical diets. Physical activity may increase the requirements for vitamins engaged in metabolic pathway leading to the ATP production and require additional multivitamin/mineral supplement (Woolf & Manore, 2006). That is the reason why vitamin B_1_ supplementation is often expressed in food per energy consumed. Daily requirement of thiamine in human ranges from 50 μg/MJ to 100 μg/MJ of diet, what corresponds to 0.6 ÷ 0.8 mg/day (males) and 0.4 ÷ 0.6 mg/day (females) (Commission of the European Communities, 1993).

The syndrome of the TD in humans is especially observed in cases of alcoholism, poor nutrition and the AIDS. In the latest case, symptoms are restricted to central nervous system. Also genetic disorders in synthesis of thiamine pyrophosphate, which is the active form of this vitamin, or the shortage of thiamine carriers transporting it into mitochondria may result in the appearance of TD symptoms (Brown, 2014; Chen et al., 2014).

The metabolic disorders seem to be associated with inability to oxidize food sources responsible of high‐caloric input. It was shown in patients undergoing coronary surgery that thiamine treatment increased oxygen consumption suggesting adequate aerobic metabolism of patients receiving thiamine (Anderson et al., 2016). Additional factors affecting bioavailability of thiamine and its phosphates, besides anti‐thiamine factors, were found, for example magnesium or pyrimidine deficiency (Baroncini, Annovazzi, Minonzio, Franzetti, & Zaffaroni, 2017; Tylicki, Łotowski, Siemieniuk, & Ratkiewicz, 2018).

The aim of this study was to investigate the effect of thiamine deficiency on glucose transmembrane transport and degradation of fatty acids in rats fed the thiamine‐deficient diet.

## 2 MATERIALS AND METHODS

### 2.1 Animals

All procedures performed in this study were in accordance with the ethical standards of the local Commission of Ethic (Warsaw, Poland). The 35‐day study was performed on 77 male Wistar rats weighting 116.6 ± 11.7 g at the beginning of the experiment. Animals were purchased from the Medical Research Center of the Polish Academy of Sciences. Rats were housed individually in transparent plastic cages with the floor made of glass rods. The rats were maintained at 22 ± 1°C and 55 ÷ 60% relative humidity, with a 12 hr light–dark cycle. Animals were given free access to food and water, and their weight gains, as well as food intake, including spills, were monitored daily. Water consumption was not measured. During five‐day adaptation period, all rats were offered standard diet (AIN‐93M) containing 6 mg of thiamine per kg diet—control diet (Reeves, 1997). After the adaptation period, 11 rats (time 0) were sacrificed. Then, remaining 66 rats were randomly divided into two feeding groups of 33 animals. The control group was offered the same diet (AIN‐93M), and the experimental one was offered the semi‐purified diet prepared from the same components as AIN‐93M diet but it was thiamine‐deficient (TD). The following components were used: casein, wheat starch, potato starch, soybean oil, premix without thiamine, L‐methionine, choline bitartrate, tert‐butylhydroquinone (TBHQ). Blood was collected on standard EDTA as anticoagulant for isolation of red blood cells (RBC) and following measurement of glucose transport. The β‐oxidation was measured in isolated leucocytes and insulin concentration in blood serum.

On 10th, 20th and 30th days, subsequent eleven rats from control and thiamine‐deficient group were sacrificed after overnight fasting, under anaesthesia with the intraperitoneal injection of thiopental sodium (Rotexmedica GmbH).

### 2.2 Analytical methods

The concentration of thiamine in the diet was determined by modified method of Leveille (1972) with the use of a photo fluorimeter (Kontron Instruments) equipped with computer software SFM 25.

Erythrocytes (RBC) and leucocytes (WBC) were separated by centrifugation of whole blood on the gradient of HISTOPAQUE‐1077. RBC formed bottom layer, while WBC were located on the top of HISTOPAQUE. Both types of cells were collected, washed twice with PBS and resuspended in initial volume of blood.

Glucose uptake by RBC was studied by the method of Lee, Yen, Shen, and Chen (2000) with the modification for different kind of cells. Erythrocytes were incubated for 15 min at 37°C with 4 mM glucose and 0.1 μCi 2‐dexy‐D‐[1,2‐^3^H] glucose (specific activity 60 Ci/mol). After the incubation, the solution with cells was centrifuged for 10 min at 1,000 g, the pellet was washed 3‐times with 2 ml of cold physiological saline solution, dissolved in 100 μl of 2 M NaOH and counted in Packard TriCarb scintillation counter. Results were presented as μmoles/g haemoglobin/hr (μmol g^−1^ Hb hr^−1^). In house estimation of variation coefficient between duplicates was in average 2.6% for 6 RBC samples.

Fatty acid degradation was examined by the method of Manning, Olpin, Pollitt, and Webley (1990) with the modification of Kuryl, Adamowicz, Debski, Bertrand, and Martynik (2001). Cells were incubated for 1 hr at 37°C with palmitic acid (52.4 nmol) and 0.1 μCi of [9, 10–^3^H] palmitic acid (sp. act. 40 ÷ 60 Ci/mol). After 2 hr of incubation, samples were precipitated with 200 μl of 10% trichloroacetic acid and 100 μl of Hank's solution was added, the samples were then centrifuged for 10 min at 2000 g. Supernatant was collected, alkalized with 100 μl of 2 M NaOH and passed through 2 cm column (0.5 cm) of Bio‐Rad AG‐1 X8 ionic exchange resin to absorb of non‐degraded palmitic acid. Effluent containing tritiated water formed from degraded substrate was counted in Packard TriCarb beta‐scintillation counter. Results were expressed as pmoles of palmitic acid decomposed by 1 mg of leucocyte protein during 1 min.

Haemoglobin was estimated by cyanmethaemoglobin method of Drabkin, glucose—by oxidase method (GLUCOSE‐OXY Kit) from Pointe Scientific Poland. Insulin concentration was assayed in blood serum using RIA Kit for rat insulin (Linco Research) containing specific monoclonal anti‐rat insulin antibody (limit of sensitivity—0.1 ng/ml).

### 2.3 Statistical analyses

For statistical evaluation of the effect of the thiamine deficiency, two‐way analysis of variance was used (group × time), SPSS 12.0pl software. Before performing analysis of variance, the equality of variances for variables was assessed using Levene's test. Homogeneity of variances was confirmed for all variables. There was a significant interaction between factors; hence, one‐way analysis of variance was also performed. Significance of difference between means was confirmed by Scheffe's and Dunnett T3 post hoc tests at *p* ≤ 0.05.

## 3 RESULTS

### 3.1 Diet and thiamine consumption

The Recommended Dietary Allowance for humans from 9 years of age and older ranges from 0.9 to 1.2 mg per day (National Academy of Sciences, 1998). The recommended dietary content of thiamine in rat diet is estimated to be about 400 μg/100 g (4 mg/kg); however, higher concentration may be required with low‐protein, high‐carbohydrate diets (National Research Council (US) Subcommittee on Laboratory Animal Nutrition 1995). The other authors estimated minimum thiamine requirement for weanling rat at 0.55 ± 0.07 mg/kg diet (Rains, Emmert, Baker, & Shay, 1997), which was substantially lower than the NRC estimated requirement of 3.1 mg/kg diet (National Academy of Sciences, 1998). The experimental diet contained 18.1 μg of thiamine per kg diet (0.018 mg/kg diet) and thus was considered as vitamin B_1_ deficient one. During 30‐day study, rats fed a thiamine‐deficient diet did not manifest neurological changes.

**Figure 1:**
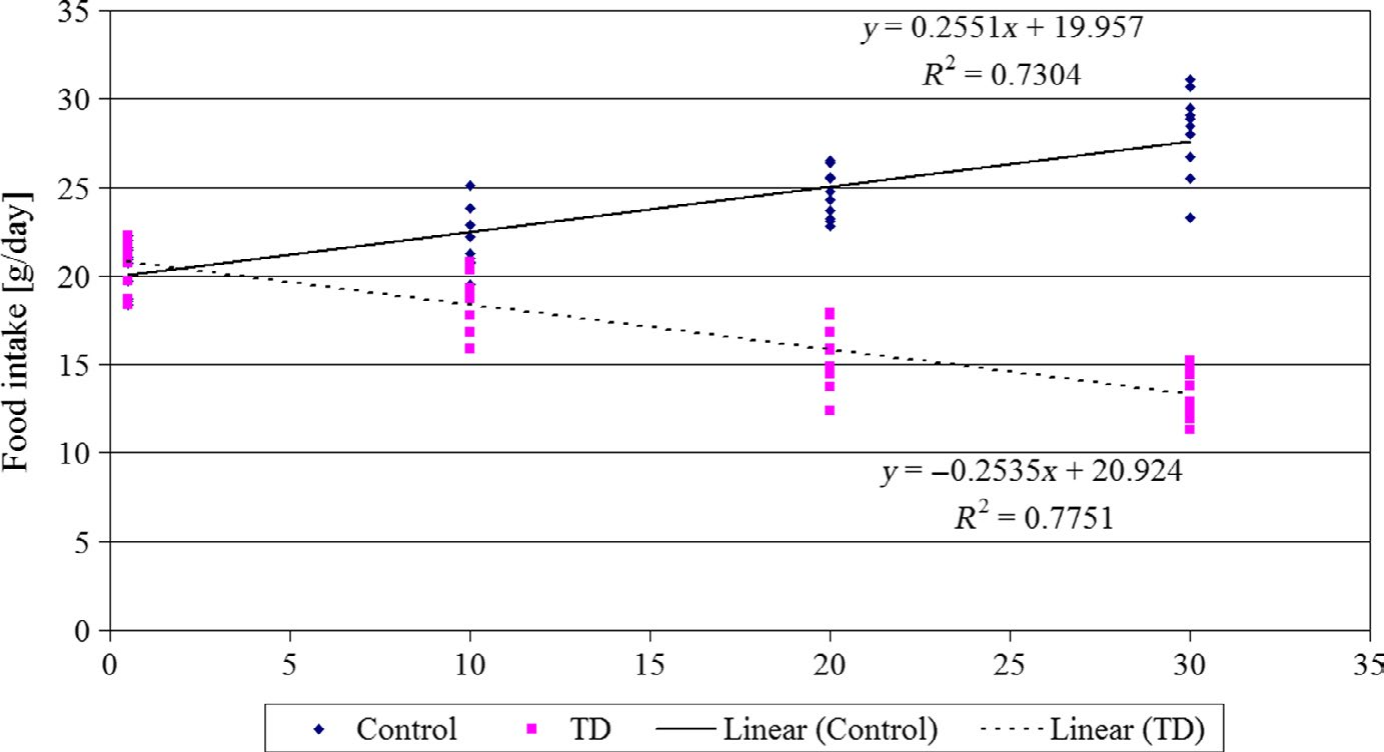
Food intake (g/day) in rats of control group and rats given thiamine‐deficient diet (TD). Duration of the experiment‐30 days, *n* = 11 [Colour figure can be viewed at http://wileyonlinelibrary.com]

The consumption of the diet by animals is presented in Figure 1. The diet consumption was 20.58 ± 1.38 at the beginning of experiment (day 0), than in the thiamine‐deficient group it was decreasing persistently to the level of 13.4 ± 1.42 on 30th day. This resulted in a very low body gain during 30‐day study (21 g). On the contrary, in the control group the consumption of the diet reached the level of 28.1 ± 2.4 g day^−1^ rat^−1^ (30th day) and about 14‐fold higher body gain (280 g) than in the control group.

A population reference intake for thiamine of 0.1 mg/MJ (0.42 mg/1000 kcal) for all population groups (European Food Safety Authority, 2016) is corresponding to about 1 mg/day (14 μg/kg b.w.). Reduced daily intake of the TD diet by experimental animals resulted in the mean thiamine intake of 2.08 ± 0.23 μg/kg body weight. The intake of thiamine in the TD group decreased significantly as compared to the recommended NRC dose, what in turn resulted in the dramatic drop of glucose uptake by erythrocytes, as well as of utilization of fatty acids by leucocytes. This decrease of the intensity of energetic processes was accompanied by a slight decrease of insulin concentration (Figure 4).

### 3.2 Glucose uptake and β‐oxidation efficiency

**Figure 2:**
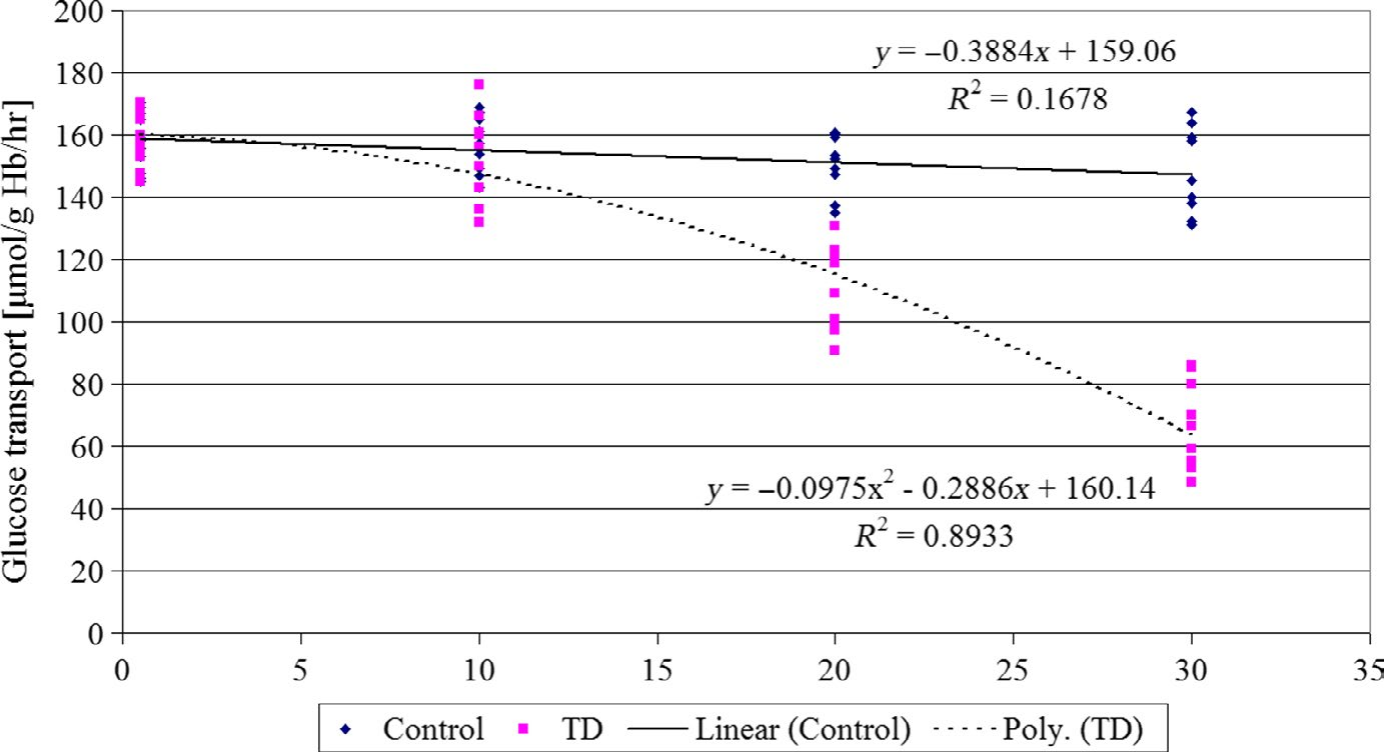
Glucose transport across the RBC membrane (µmol g^−1^ haemoglobin hr^−1^) of control rats and rats given thiamine‐deficient diet (TD). Duration of the experiment‐30 days, *n* = 11 [Colour figure can be viewed at http://wileyonlinelibrary.com]

Glucose uptake by erythrocytes was dropped from 158.03 ± 8.77 μmol g^−1^ Hb hr^−1^ at the beginning of the experiment to 65.87 ± 14.03 μmol g^−1^ Hb hr^−1^ after 30 days of feeding of rats with thiamine‐deficient diet (Figure 2). Differences between control rats and group of rats receiving the TD diet were significant on 20 and 30 days of experiment. In control group of rats, no significant changes in glucose transport across RBC membranes were observed.

**Figure 3:**
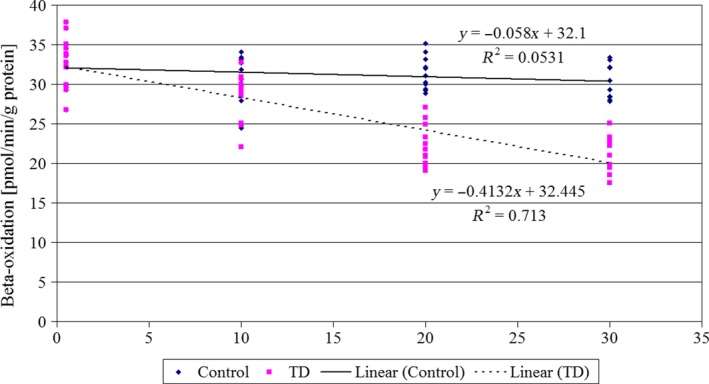
The β‐oxidation of fatty acids (pmol min^−1^ mg^−1^ protein) in leucocytes of control rats and rats given thiamine‐deficient diet (TD). Duration of the experiment‐30 days, *n* = 11 [Colour figure can be viewed at http://wileyonlinelibrary.com]

In thiamine‐deficient rats, respective values for β‐oxidation (Figure 3) were 32.67 ± 3.17 (day 0) and 21.21 ± 2.24 pmol min^−1^ mg^−1^ leucocyte protein (day 30). Drop of β‐oxidation in rats receiving thiamine‐deficient diet was statistically significant since 10 days of experiment.

### 3.3 Insulin concentration

**Figure 4:**
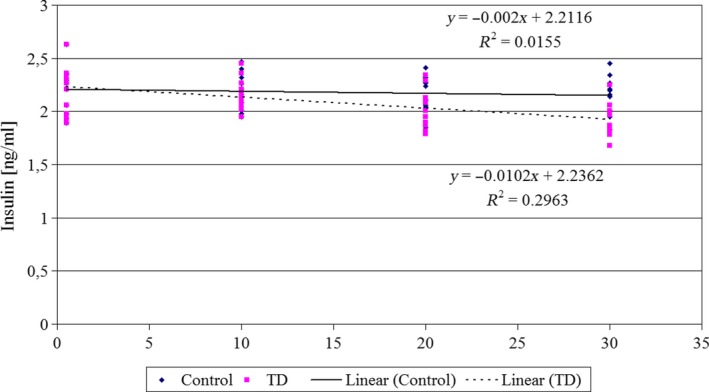
Concentration of serum insulin (ng/ml) in rats of control group and rats given thiamine‐deficient diet (TD). Duration of the experiment ‐ 30 days, *n* = 11 [Colour figure can be viewed at http://wileyonlinelibrary.com]

Thiamine deficiency exhibits slight effect on insulin level in serum (Figure 4). During 30 days of the experiment, insulin level dropped from 2.22 ± 0.21 (day 0) to 1.93 ± 0.16 ng/ml (day 30th). This decrease was very small but the application of the Dunnett T3 post hoc test proved its significance (*p* = 0.045), as compared to the day 0.

## 4 DISCUSSION

Thiamine deficiency (TD) led to the significant reduction of the feed intake (Figure 1) in growing rats. It contributed to energy deficiency which resulted in retardation of growth in young rats.

In human, the loss of appetite is often the presenting symptom of TD and should be regarded as a protective phenomenon, since continued intake of a high‐carbohydrate diet could aggravate the deficiency. Liu et al. (2014) showed in mice that thiamine deficiencyinduced anorexia. Our results confirmed the previous findings that thiamine plays an important role in regulation of food/feed consumption and body weight programming (Ba, 2012). In the experiments performed on laboratory animals, significant drop of diet consumption and growth of animals fed a TD diet was also observed previously (Klooster et al., 2009; Liu et al., 2014). Thiamine re‐supplementation of TD animals restored their appetite which resulted in body weight recovery after seven days (Liu et al., 2014). However, the other authors stated that thiamine deficiency during perinatal period significantly reduced birth weight of rats comparing with per‐fed controls and this growth retardation persisted in adulthood (Ba, 2012; Balaji et al., 2015; Ozdemir et al., 2018).

Thiamine deficiency (TD) lowered glucose uptake by erythrocytes, not immediately but with a certain delay. Glucose transmembrane transport was resistant to vitamin B_1_ deficiency up to 10 days of the experiment, then significantly decreased to 42% of the initial value on day 30 (Figure 2). These results are in agreement with the observations of Sang et al. (2018) who correlated the TD with brain glucose hypometabolism in Alzheimer's disease. The low dietary level of thiamine caused a suppression of ATP synthesis (to 70% of the control value) and lowered phosphocreatine levels in different brain structures of rats (Aikawa et al., 1984). To maintain adequate aerobic metabolism in any tissue, vitamin B_1_ is indispensable. Thiamine deficiency inhibited oxidative processes in glycolysis and Krebs’ cycle and resulted in accumulation of reduced NADH + H^+^ and FADH_2_, and acidic intermediates of glycolysis—pyruvic and lactic acids (Brin, Shohet, & Davidson, 1958; Pannunzio, Hazell, Pannunzio, Rao, & Butterworth, 2000). Thiamine deficiency caused also 20% loss of activity of enzymes necessary for oxidation processes: pyruvate decarboxylase and α‐ketoglutarate dehydrogenase in rat liver (Blair et al., 1999). Giacalone et al. (2015) presented results of three patients receiving parenteral nutrition without thiamine supplementation. The thiamine deficiency caused a severe lactic acidosis, but i.v. thiamine administration resulted in rapid reversal of observed acidosis.

Activity of fatty acids degradation diminished from day 0 to day 20 by 35% and the further decrease was much slower (Figure 3). It was found that a diet composition may have significant influence on β‐oxidation of fatty acids. In our previous experiment on rats subjected to thiamine deficiency (Debski, Kuryl, Gralak, Pierzynowska, & Drywien, 2011), it was noticed that the supplementation of the TD diet with non‐digestible fructo‐oligosaccharides ameliorated the inhibition of β‐oxidation caused by dietary thiamine deficiency. Results concerning β‐oxidation activity (oxidation at second carbon atom and liberation of acetyl‐CoA from fatty acid molecule) are similar to those for α‐oxidation (oxidation at first carbon atom and liberation of CO_2_) of fatty acids, reported by other authors (Depeint, Bruce, Shangari, Mehta, & O’Brien, 2006; Fraccascia, Sniekers, Casteels, & Veldhoven, 2007; Sniekers et al., 2006). Observed suppression of palmitic acid β‐oxidation was higher than those reported by Sniekers et al. (2006) for α‐oxidation of palmitic acid and phytanic acid. However, our assays were done after 20 and 30 days of thiamine deprivation while Sniekers’ group—after only 5 days.

Inhibition of β‐oxidation of fatty acids was accompanied by lowering of serum insulin concentration by 13% in the period 0–30 days of this experiment. Ratahanaswami et al. (1991) showed in in vitro studies on pancreatic islets taken from thiamine‐deficient rats, that stimulation of insulin secretion by glucose, leucine or arginine was inhibited. In our experiment, a decrease of the insulin concentration in blood serum was only moderately correlated to glucose transportation (*r* ^2^ = 0.23) and β‐oxidation (*r* ^2^ = 0.17). This may suggest that in case of thiamine deficiency, there are other factors, besides insulin, affecting metabolism of sugars and fats. In early stages of thiamine deficiency, glucose metabolism was less sensitive as compared to fatty acids degradation. However, prolonged deficit of vitamin B_1_ resulted in the fast decrease of glucose uptake, while further decrease of β‐oxidation slowed down.

In animals, which exhibit improper transmembrane transport of glucose, activity of ATP synthesis from sugars degradation must be low, and it should be, at least partially, restored by increased β‐oxidation of fatty acids. However, in the absence of thiamine, which is necessary for proper activity of several dehydrogenases, for example pyruvate dehydrogenase or reduced nucleotides dehydrogenases, the efficiency of fatty acids degradation is lower than the level of fatty acids oxidation in control animals fed the diet containing recommended doses of thiamine at the beginning of experiment.

## 5 CONCLUSIONS

1. Dietary thiamine deficiency suppresses the food intake in animals.
2. Glucose uptake by cells and β‐oxidation of fatty acids are inhibited in rats fed thiamine restricted diet, but are not significantly correlated with serum insulin level.
